# An Objective Scatter Index Based on Double-Pass Retinal Images of a Point Source to Classify Cataracts

**DOI:** 10.1371/journal.pone.0016823

**Published:** 2011-02-04

**Authors:** Pablo Artal, Antonio Benito, Guillermo M. Pérez, Encarna Alcón, Álvaro De Casas, Jaume Pujol, José M. Marín

**Affiliations:** 1 Laboratorio de Óptica, Universidad de Murcia, Murcia, Spain; 2 Hospital Universitario Virgen de la Arrixaca, Murcia, Spain; 3 Centre de Desenvolupament de Sensors, Instrumentació i Sistemes (CD6), Universidad Politécnica de Cataluña, Tarrasa, Barcelona, Spain; The University of Hong Kong, Hong Kong

## Abstract

**Purpose:**

To propose a new objective scatter index (OSI) based in the analysis of double-pass images of a point source to rank and classify cataract patients. This classification scheme is compared with a current subjective system.

**Methods:**

We selected a population including a group of normal young eyes as control and patients diagnosed with cataract (grades NO2, NO3 and NO4) according to the *Lens Opacities Classification System* (LOCS III). For each eye, we recorded double-pass retinal images of a point source. In each patient, we determined an objective scatter index (OSI) as the ratio of the intensity at an eccentric location in the image and the central part. This index provides information on the relevant forward scatter affecting vision. Since the double-pass retinal images are affected by both ocular aberrations and intraocular scattering, an analysis was performed to show the ranges of contributions of aberrations to the OSI.

**Results:**

We used the OSI values to classify each eye according to the degree of scatter. The young normal eyes of the control group had OSI values below 1, while the OSI for subjects in LOCS grade II were around 1 to 2. The use of the objective index showed some of the weakness of subjective classification schemes. In particular, several subjects initially classified independently as grade NO2 or NO3 had similar OSI values, and in some cases even higher than subjects classified as grade NO4. A new classification scheme based in OSI is proposed.

**Conclusions:**

We introduced an objective index based in the analysis of double-pass retinal images to classify cataract patients. The method is robust and fully based in objective measurements; i.e., not depending on subjective decisions. This procedure could be used in combination with standard current methods to improve cataract patient surgery scheduling.

## Introduction

Intraocular scattering may severely degrade the retinal image quality [1]. In eyes suffering early cataracts symptoms this can produce visual disturbances, even if visual acuity remains normal [2]. This has important practical implications; for example the optimal scheduling of a cataract surgery should be determined by the moment when the visual impairment produced by the cataract affects the normal daily activities [3]. The subjectivity implicit in this definition has stimulated the development of a variety of methods for classifying cataracts, that may permit a somehow standardized procedure. However, until today there is no consensus on the most adequate technique to predict the visual disturbances associated with a gradation of cataracts.

A first group of methods to evaluate cataract is composed by those based on the analysis of slit-lamp pictures of the patient's crystalline lens. The Lens Opacities Classification System III (LOCS III) [4] is one of the most representatives. Although the analysis considers different aspects like color, grade of opacification or cataract type, the final decision strongly depends on the subjective criterion of the clinician. In addition, the slit-lamp images only provide information related to the back-scattered light, while actually is forward scatter, the responsible of the degradation of retinal image in cataract patients [5]. On other hand, the relationship between the backward and the forward scatter produced in the crystalline lens depends on the type of cataract [6], which increases the difficulty of a direct interpretation of the slit-lamp images to evaluate the detrimental effect of the cataract on visual performance.

There are procedures based on the objective analysis of the image of the crystalline lens acquired either with a standard slit-lamp device [7], or with a Scheimpflug camera [8]. More recently, a method based on the analysis of the dynamic light scattering of the crystalline lens has been proposed [9]. All these techniques evaluate the development of the cataract process by the analysis of the image of the crystalline lens, but they do not directly consider the actual degradation of the retinal image and the associated impairment on the visual performance.

There is a second group of methods based on the evaluation of the degradation produced by the cataract on different visual functions. The evaluation of visual acuity or contrast sensitivity in the presence of a glare source [10], [11] can be considered as classical examples of these methods. Other approaches are based on a more specific analysis of the contribution of the intraocular scattering. One of these systems is the C-Quant instrument (Oculus GmbH, Wetzlar-Dutenhofen, Germany) that uses the compensation-comparison method [12] to evaluate intraocular scattering [13], [14]. These types of procedures capture the impact of forward scatter in vision, but since they are subjective, the active participation of the patient is required.

We propose here a new optical method to estimate the contribution of forward scattering that affects vision to characterize the severity of cataracts. The method is based on the analysis of the double-pass (DP) image of a point source projected on the retina. The DP technique has been widely used in the past to evaluate the optical quality of the retinal image [15], [16] for a variety of conditions [17]–[19]. A different analysis of the light distribution in the DP image has been developed to quantify intraocular scattering [20], [21]. The procedure has been applied in a control group and in eyes with different scatter severity to demonstrate its validity as a predictor of the effect of cataracts.

## Methods

### Subjects

We tested the procedure in 53 eyes: 15 corresponding to a group of normal young subjects (average age = 28±5 y/o); and the other 38 eyes (average age = 73±7 y/o) were patients with diagnosed cataracts. One eye per patient was evaluated. Every patient was informed of the subject of the study, and a written informed consent obtained, following the tenets of the Declaration of Helsinki. The study protocol was approved by the “Hospital Virgen de la Arrixaca” ethics committee. Firstly, we evaluate the subjects' visual acuity and refraction. In every subject, an ophthalmological exam was completed, including Optical Coherence Tomography (OCT) measurements to discard macular problems and the recording of images of the crystalline lens provided by a slit-lamp device, after dilating the pupil by instilling 0.2 ml of tropicamide (1%).

Inclusion criteria for the group of cataract patients was nuclear cataract with increasing lens opacity, although not all cases were purely nuclear cataracts, as some subjects also showed slight degree of capsular opacification. Every patient was classified according to the degree of nuclear opacification (NO), by using the LOCSIII chart [4], following the subjective decision of the ophthalmologists participating in the study. The 38 cataract eyes were classified as follows: 12 eyes as grade 2 (NO2), 18 eyes as grade 3 (NO3), and 8 eyes as grade 4 (NO4).

### DP images acquisition and processing: Objective Scatter Index

**Figure 1:**
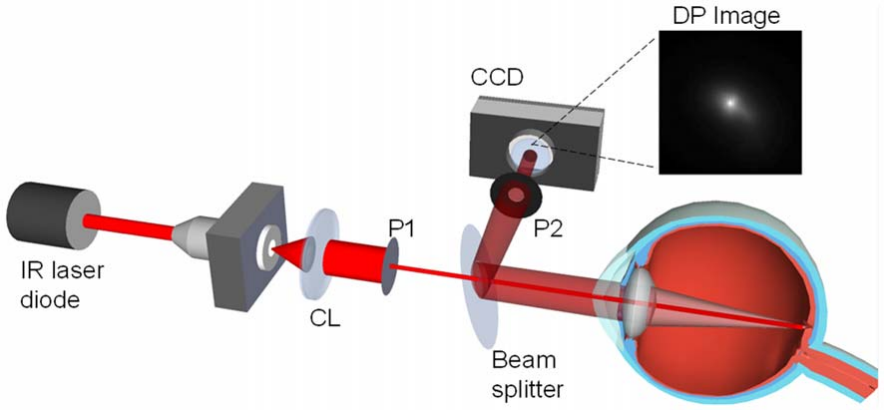
Schematic representation of the double-pass method. See text for further details.

We used a clinical double-pass instrument (OQAS II, Visiometrics S.L., Tarrasa, Spain) to acquire the DP images. This DP system is based on an unequal pupil configuration [22] with a diameter of 2-mm in the entrance pupil and a variable diameter for the exit pupil. In this study, the diameter of the exit pupil was set to 4 mm for the whole procedure. Fig. 1 shows a schematic representation of the instrument. An infrared diode laser is collimated (CL) and after passing the entrance aperture (P1) enters the eye. After reflection in the retina and double-pass through the ocular media, the light is reflected in a beam splitter and passes the exit aperture (P2) to be recorded in a digital camera. We acquired DP images of every eye at best focus, corrected internally by the instrument by an optometer which ranges from −8D to +6D. The astigmatism was also corrected by using the appropriate cylindrical lens on a holder placed in front of the eye.

We recorded series of images from every eye assuring that the peak intensity level was above 200 (as a maximum value of 255) and background images, with a light trap in place of the eye. Both, the series of DP retinal images and the background images were averaged, and a final image was obtained for each eye as the subtraction of the mean background from the mean DP image.

From each image, we defined an Objective Scatter Index (OSI) to quantify the magnitude of the intraocular scattering. It is based on the analysis of the intensity distribution in the outer parts of the DP image. The parameter OSI is defined as the ratio between the integrated light in the periphery and in the surroundings of the central peak of the DP image. The eccentricity and width of the specific areas for this calculation depends on the acquisition parameters of the DP system. In the particular case of the instrument OQAS, the central area selected was a circle of a radius of 1 minute of arc, while the peripheral zone was a ring set between 12 and 20 minutes of arc. The extension of the areas considered imposes a limit for a hypothetical eye with maximum scatter (i.e.; a uniform distribution of intensity on the image). A multiplying factor of 0.1 limits the accessible values of OSI from 0 to a maximum of 25. OSI for normal eyes would range around 1, while values over 5 would represent highly scattered systems.

**Figure 2:**
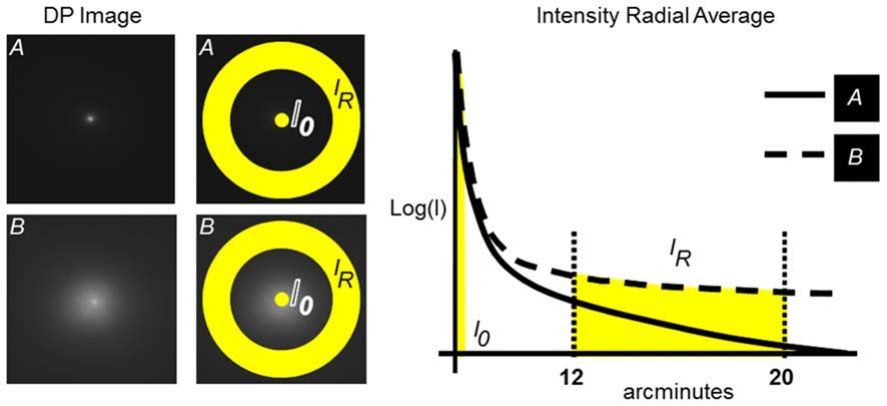
Example of the selection of the zone in the double-pass image used to define the objective scatter index (OSI). We compute the relation between the intensity in the peak of the image, the central area within 1 minute, with that in a ring comprised between 12 and 20 minutes. Two DP images with different levels of scatter (A less scatter, B more scatter) are represented to illustrate the procedure.

Fig. 2 shows two examples on how the 2D double-pass retinal images are converted into 1D intensity profiles as radial averaged and the areas selected to integrate the intensity. A system with more scatter (example image B in figure 2) will produce an image with higher intensity in the peripheral areas and then an elevated value of OSI.

### Impact of aberrations in the Objective Scatter Index

The retinal image is affected by both ocular aberrations and intraocular scattering [23], [24]. These two factors influence the light distribution on the retina. While the ocular aberrations influence the light distribution at the central region of the image, the intraocular scattering cause an increment of the intensity further away from the central maximum of the retinal image, leading to a wide angle halo. The effect of the combination of both factors on the visual quality is not a simple summation of the degrading effects since, as it has been recently discovered, the addition of spherical aberrations improved contrast in the presence of scatter [25].

The interpretation of the OSI as a parameter related to the intraocular scattering requires an evaluation of the contribution of ocular aberrations on this index. It may be possible that the presence of aberrations could contribute to the outer areas of the DP images affecting the OSI values. There is a trade-off between the area registered within the DP and the amount of aberrations that would affect the parameter. We addressed this issue by comparing experimental DP images with simulated ones, calculated from actual data of the aberrations measured in the same eye using a Hartmann-Shack (HS) wave sensor [26].

**Figure 3:**
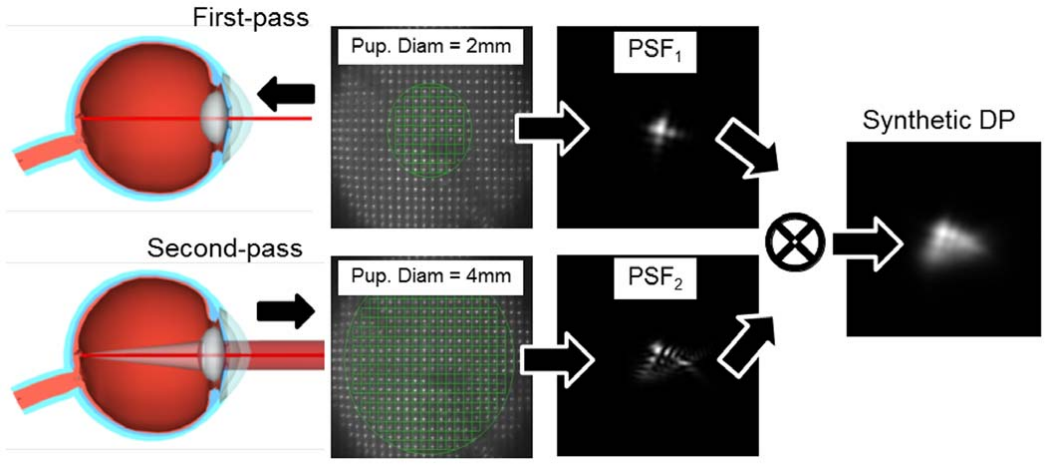
Schematic representation of the procedure to compute the synthetic DP image from wavefront aberration data. We computed the PSF for a 2-mm (first-pass) and 4 mm (second-pass) pupil diameter. Convolution of both PSFs renders the synthetic DP image.

**Figure 4:**
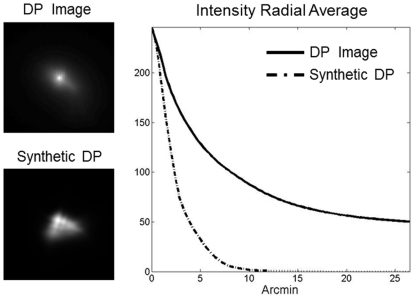
Example of comparison of actual and synthetic DP images. The 1D section shows the range where scatter is significant and used to calculate the OSI parameter.

Fig. 3 shows a schematic representation of the procedure to compute a synthetic DP image by using exclusively the information provided by the HS images (related only to aberrations). The DP image is the convolution of the point-spread function (PSF) corresponding with the first and the second passes [15]. From the HS images recorded for every analyzed eye, we obtained the wave aberrations and the PSF of the incoming pass (PSF1) and the PSF of the outgoing pass (PSF2) were calculated. Finally, the synthetic DP image is the result of the convolution between these two PSFs. The synthetic DP image can be compared with the experimental DP for the same eye. They should be very similar in eyes with small amount of scatter (the optical quality dominated by aberrations) and different in eyes severely affected by scatter. Fig. 4 shows an example of both, the measured DP and the calculated from the aberrations, together with a radial averaged section for each case. The results if this figure justifies the selected area (12–20 minutes of arc) that affect scatter to calculate the OSI parameter.

**Figure 5:**
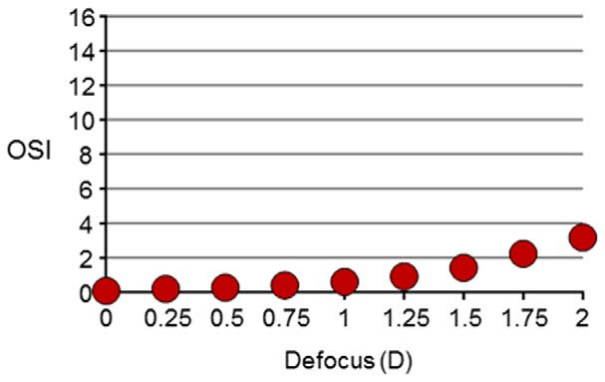
Estimated value of OSI as a function of induced defocus. Uncorrected refractive errors over 1 diopter induce artifacts ion the determination of OSI over the value of 1 (standard value in normal eyes). This indicates that refractive errors need to be well corrected during the DP data collection to provide accurate values of OSI. The y-axis is kept scaled to16 as in the figures where OSI is represented. This will permit a direct comparison on the impact of uncorrected defocus on OSI.

From a practical perspective, in addition to high order aberrations, the OSI parameter could also be largely affected by uncorrected refractive errors (defocus and astigmatism). To define the acceptable range of uncorrected defocus, we recorded a series of DP images in a young eye for different amounts of induced defocus from 0 to 2.0 D. The calculated OSI as a function of defocus is shown in Fig. 5. For uncorrected values over 1.0 D, significantly large OSI values (more than 1) are obtained. To avoid this type of artifact in the OSI determination, the DP images need to be recorded with both, defocus and astigmatism, corrected at least with a precision better than 1.0 D of spherical equivalent.

## Results

**Figure 6:**
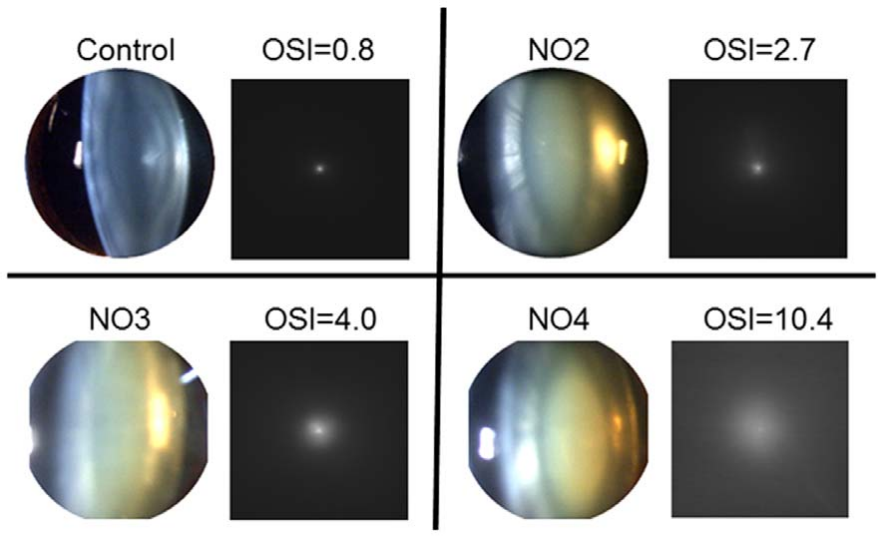
Example of DP and slit-laps images in four eyes as example of the four groups. The actual OSI value in each eye is noted.

**Figure 7:**
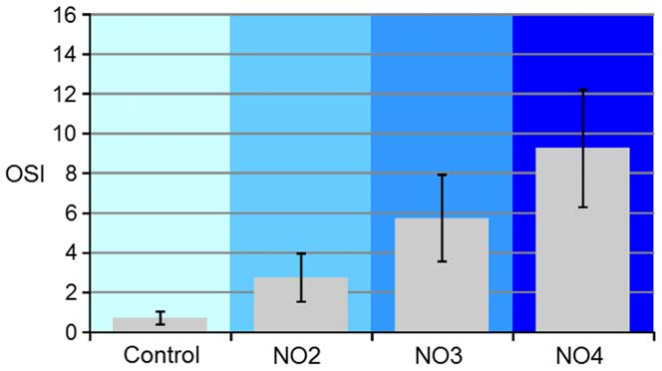
Average OSI for the four groups analyzed: control group (left), and groups of eyes with opacification according to the LOCSIII chart classification (groups NO2, NO3 and NO4). The error bar represents standard deviation.

The OSI parameter was estimated from the DP images obtained for the 53 eyes included in the study. Figure 6 shows, as an example, DP and slit-lamp images in four representative eyes from each group: control and NO2-NO4. DP images are clearly deteriorated for increasing levels of scatter. We calculated the average values of OSI in the patients following the LOCSIII classification. Figure 7 shows the average values of OSI for each group: 0.7±0.3 for the control group; 3.0±1.0 for the NO2 group, 6.0±2.0 for the NO3 group, and 9.0±3.0 for the NO4 group. Although the OSI values clearly increased following the subjective classification, there was a significant dispersion especially in groups NO3 and NO4.

**Figure 8:**
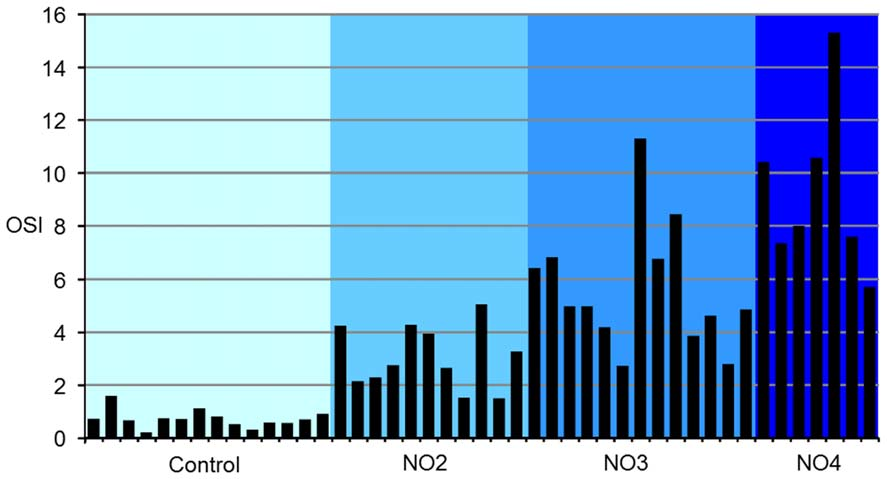
Individual OSI values for every tested eye. Normal young eyes (Control; left) showed small values of OSI. The eyes in groups NO2, NO3 and NO4 showed increasing OSI values, although with clear individual variability.

Figure 8 shows the individual values of OSI in all tested eyes. While the normal young eyes (left) showed small and similar OSI values (typically below 1), in the case of the cataract eyes, in some individuals we found OSI values either lower or higher than the average value for the corresponding group. For example, an eye classified as NO4 (the last sample right in the panel) had an OSI value similar to other eyes that were classified as NO2.

**Figure 9:**
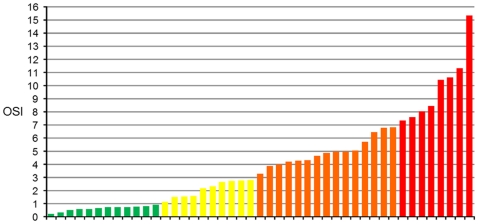
Subjects classified according to obtained OSI values. Increasing grey scale represents the gradation proposed by the quantification of cataracts, according to this objective parameter.

**Figure 10:**
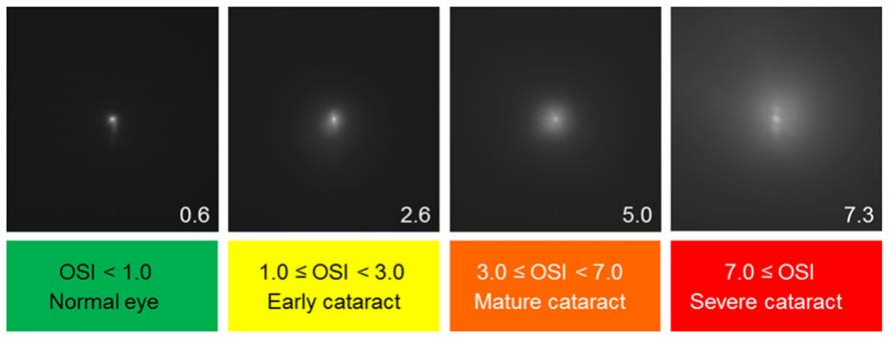
Example of DP images for the different range following the OSI classification scheme. Double-pass (DP) images from four eyes and corresponding OSI values. Below, proposed classification ranges of eyes according to the measured OSI values.

By using the measured OSI values, we are able to establish a new classification of the eyes with different degrees of intraocular scattering (Figure 9). Following this scale, the patients could be separated out in different groups: the first range (white bars) with an OSI below 1 corresponds to eyes with low amounts of scatter that can be considered as normal eyes. The next range (light grey bars) included eyes with an OSI between 1 and 3, corresponding to older eyes with associated scatter of an early cataract. The third range with OSI values between 3 and 7, corresponds to developed cataracts that should undergo surgery. Finally, eyes with severe cataracts and large amount of associated intraocular scattering (dark grey bars) presented values of OSI higher than 7. Figure 10 shows as an example DP images representative of the four ranges with the actual OSI value for each case.

## Discussion

Westheimer and Liang [27] proposed to analyze the intensity distribution of the DP images to extract information about the combined contribution of the ocular aberrations and the intraocular scattering on the retinal image. In a later work, they combined the information of the DP images with subjective measurements to separate out the influence of the intraocular scattering on the visual performance [28]. However, they did not evaluate the contribution of the ocular aberrations on the DP images. In addition, in those studies the procedure was not applied in eyes where scatter was the main degrading effect.

We have further refined this approach to evaluate the contribution of scatter separated from refractive errors and higher order aberrations, with the main intention to classify the degree of cataracts. The main consequence of the definition of the scale of values of OSI is that we can establish a comparison between the particular positions of any of the analyzed eyes in this classification and the position of the same eye in the gradation made by the LOCSIII system. This analysis would give a percentage of agreement regarding to the number of eyes classified in equivalent levels by the two scales.

It should be noted the significant advantages of the combined use of both approaches. OSI gradation directly relates with the visual degradation (forward scatter), while the information in the slit-lamp images is useful to understand the nature and type of the cataract, although it is not always simple to predict the actual visual impact. The DP image is affected by both the forward and the backward scattering, produced in the first and the second pass of the light through the crystalline lens; the analysis of the energy distribution on this image reveals the contribution of the light scattering which really impairs visual performance.

We found an agreement of 75% by considering the complete population between the two schemes (OSI versus LOCSIII). This means that 40 of the 53 eyes were sorted as equivalent by using both methods. Although this percentage is relevant, in the daily clinical practice the capability of detecting an early cataract is especially useful and therefore, it would be very interesting to show the agreement between the eyes ranked in one of the two first levels in both scales. The percentage of agreement between the eyes classified in one of the two first levels in both scales was 84%. This means that 21 of the 25 eyes, which were ranked by the values of OSI on the first and second levels, were classified accordingly to the LOCSIII chart. This further emphasizes that a value of OSI lower than 1 characterizes a non-cataract eye, whereas a value of OSI around 2 would correspond with an early cataract eye, corresponding to NO2 in the LOCSIII chart. With regard to the eyes classified in the third and fourth level of the OSI scale, they were all mature cataracts. In this case, the disagreement between LOCSIII and the OSI rankings was not quite relevant since, from a practical point of view, it does not affect the diagnosis capability of early cataract process.

The correspondence between the classification made by clinicians, by the LOCSIII chart, and the gradation established from the values of OSI, supports the applicability of this objective method to make a sound decision on the proper timing for a cataract surgery. Moreover the quantification provided by an objective gradation as OSI, can be also very useful to complete the available data from anamnesis, or reduced visual acuity in the diagnosis of an early cataract process.

In addition, it would be important to evaluate the values of OSI in other types of lens opacification, as well as to refine the possible ranges for classifying these cataracts depending on the intraocular scattering measured by OQAS. Having new data about intraocular scattering in cataracts may also be helpful to understand the developing process on cataracts, as well as to better understand the way the crystalline lens evolves from being transparent to increased dispersion as well as other morphological and optical changes, as could be the increased aberrations with age [29].

Patients mainly affected by nuclear cataract were primarily selected among a large number of potential candidates by using slit-lamp images. However, some concomitancy between nuclear with cortical or capsular cataracts is not possible to avoid. Different types of cataracts may have a different contribution on the ocular scattering. The impossibility for estimating whether there was or not some contribution of other type of cataract together with the predominant nuclear type, could be considered as one of the factors to explain the differences between the gradation made by the LOCS and the objective classification based on OSI.

The DP image, and therefore the scatter index, is affected by the complete eye. It would be possible in some particular cases to measure an elevated OSI due to corneal haze, which is not related to cataract. In the DP instrument, the laser beam in the first pass has a small diameter (1.5 mm diameter) and reaches the eye within the pupil center area. With a localized cataract the actual impact location of the beam could affect directly the image in the retina. In the second pass, after retinal reflection, the situation is different since the light fills the whole area of the pupil, and hence, the entire crystalline lens (within the area of the pupil) is considered. DP image actually contains information from the whole pupil area. However, some minor variability in the results could be observed due to the impact of the first pass. In an instrument with a large beam also in the first pass, this problem could be avoided. We should emphasize that the reported method provides average information within the complete pupil area; i.e, it is not spatially resolved. This technique, although useful, should be combined with complementary techniques routinely. The DP images were recorded using near infrared light. This is an advantage for the patient's comfort during image acquisition. However, the magnitude of scatter in infrared light can be different than in visible light. The impact of retinal scatter could be also larger in infrared since light penetrates deeper in the retina. This is a limitation for the absolute characterization of scatter, but not very relevant for the relative gradation of cataract of this study.

The changes in the cornea and the vitreous as a function of age may also increase the overall amount of ocular scattering. However, in non pathological cases, the magnitude of these effects would be smaller than that of the lens in early cataract.

In conclusion, a new objective optical method has been developed to quantify the degree of cataract. It is based on the recording and processing of double pass retinal images of a point source. We demonstrated the potential of the technique in a group of cataract patients. The correlation of the cataract gradation between this approach and a standard subjective method (LOCSIII) is significant.

However the higher sensitivity and the intrinsic nature of this approach to detect forward scattering renders our method more powerful specially in the detection of earlier stages of cataract and to relate cataract symptoms with visual complaints. The method is robust and fully based on objective measurements; i.e., not depending on subjective decisions. This procedure could be used in combination with standard current methods to improve cataract surgery patients scheduling.
